# Postoperative anaesthesia follow-up via telemedicine after outpatient surgery: a proof-of-concept study

**DOI:** 10.1186/s13037-026-00514-x

**Published:** 2026-10-15

**Authors:** Benedikt Ludwig Huerter, Rolf Rossaint, Jan Larmann, Michael Czaplik, Andreas Follmann

**Affiliations:** 1 Department of Anaesthesiology, Faculty of Medicine, RWTH Aachen University, Pauwelsstraße 30, 52074 Aachen, Germany; 2 Docs in Clouds TeleCare GmbH, Vaalser Straße 460, 52074 Aachen, Germany

**Keywords:** Telemedicine, Outpatient surgery, Postoperative visit, Patient-reported outcome measure, Peri-operative quality management

## Abstract

**Background:**

Postoperative anaesthesia visits after inpatient procedures guarantee a comprehensive assessment of the patient’s status. As the number of outpatient surgical cases in hospitals increases, more patients are recovering at home. Telemedicine may provide an efficient and effective link between anaesthesiologists and patients at home without restricting their freedom and self-determination.

**Methods:**

After an initial acceptance survey, a single-arm, observational pilot study piloted an optional postoperative anaesthesia visit for patients undergoing outpatient surgery under general anaesthesia. It was implemented using videochats and patient-reported outcome measure (PROM) questionnaires.

**Results:**

Of the 300 people initially surveyed in the initial acceptance survey, nearly two-thirds (62.0%) expressed interest in using videochats to contact an anaesthesiologist after discharge. One hundred patients (*n* = 100) were included in the subsequent observational pilot study. The offer of an optional postoperative visit was used by 65 participants (*n* = 65, 65%). Videochats were considered a reliable tool for communicating medical issues and perceived as superior to telephone calls. The use of PROM questionnaires was considered meaningful by more than 80% of participants. Sixty-two of the sixty-five participants considered the postoperative anaesthesia visit a useful and beneficial part of the treatment and recovery process.

**Conclusions:**

Postoperative anaesthesia visits via videochat combined with PROM questionnaires after outpatient surgery were successfully piloted and consistently perceived positively by participants. Telemedicine-assisted postoperative anaesthesia visits may be a feasible component of patient-centred care in settings with limited resources.

**Trial registration:**

German Clinical Trials Register; https://drks.de/search/en; registration number: DRKS00028755, registration date: 26th January 2023.

## Highlights

The standard practice of postoperative anaesthesia visits can be challenging in outpatient procedures, for example due to physical distance.

Using videochat and PROMs, we piloted an optional postoperative anaesthesia visit that also takes into account aspects of efficiency and patient autonomy.

Postoperative anaesthesia visits can be implemented after outpatient procedures and can be a feasible means to provide efficient, individualized patient support without the need for in-person visits.

## Background

With more than 1.6 million surgical procedures covered by statutory health insurance annually, outpatient surgery in hospitals has become increasingly popular in Germany in recent years following the trend of other countries with even higher case numbers [1]. Outpatient treatment allows patients to recover in a familiar environment and minimizes the time they spend in the hospital. Recovery is usually largely self-determined. The extent of the surgery does not determine the extent of expected postoperative complications. Outpatient procedures can result in a level of pain that requires intervention [2].

Assessment of patients’ postoperative status has evolved from measuring morbidity and mortality to a more multifaceted assessment of the quality of anaesthesia and surgery, including patient satisfaction [3]. To assess patients’ status and treat complications like pain, postoperative anaesthesia visits are defined as a fundamental part of anaesthesia care [4]. This physician-patient contact highlights the peri-operative role of anaesthesiologists and supports constant reflection on quality [5]. However, the effort for postoperative visits is reportedly high [6]. Outpatient procedures are particularly challenging because of the physical distance and limited contact possibilities. Nevertheless, symptoms experienced after discharge may require medical consultation [2]. Telemedicine establishes location-independent interaction methods and can be an efficient and effective link to healthcare access [7].

During the patient’s time in the hospital, surgeons and anaesthesiologists jointly assess patients’ status and decide on matters such as the appropriate time for discharge [8]. Following outpatient procedures, the largely self-determined recovery process involves limited access to medical evaluation. Patient-reported outcome measures (PROMs) such as the Quality of Recovery-15 (QoR-15) enable patient-centred assessments without direct contact to a physician [9, 10]. The European Society of Anaesthesiology has recommended the QoR-15 for use in clinical practice [11]. PROM questionnaires have been shown to be valid and reliable for outpatient settings [12]. Consequently, the combination of telemedicine and PROMs could support anaesthesiologic patient care while not limiting patients’ freedom and self-determination.

The objective of the observational pilot study was to implement and evaluate a postoperative anaesthesia visit via videochat combined with PROM questionnaires after outpatient procedures.

## Methods

### Study design and ethics

To assess the need for and acceptance of telemedicine-assisted postoperative anaesthesia visits from the patient’s perspective, an anonymous initial acceptance survey was conducted at the Outpatient Surgery Centre (OSC) of the University Hospital of RWTH Aachen, Germany, from September 2020 to July 2021.

Following this, a single-arm observational pilot study was conducted at the Outpatient Surgery Centre (OSC) and Department of Anaesthesiology, University Hospital of RWTH Aachen, Germany, from February to August 2023. Using videochats and PROM questionnaires, we offered postoperative anaesthesia visits after outpatient surgery. Ethical approval for the studies (Ethical Committee N° EK 21–076 and N° EK 22–302) were provided by the Ethical Committee of University Hospital Aachen, Pauwelsstrasse 30, 52,074 Aachen, Germany (Chairperson Prof G. Schmalzing). The observational pilot study was registered in the German Clinical Trials Register (DRKS00028755) which is also published on the WHO Trials Portal on 26th January 2023. The procedures followed were in accordance with the latest version of the Declaration of Helsinki.

### Participants

For the initial acceptance survey, German-speaking patients of legal age ( > = 18 years) who had undergone general anaesthesia for outpatient surgery at the OSC were invited to participate shortly before discharge.

For the observational pilot study, German-speaking patients of legal age ( > = 18 years) who were scheduled for outpatient surgery under general anaesthesia at the OSC were contacted by the study team before their pre-operative anaesthesia evaluation at the Department of Anaesthesiology and asked for participation. To be eligible for participation, patients were required to have access to an internet-compatible device with speakers, microphone and camera e.g. a laptop, tablet or mobile phone. Patients were informed that participation was entirely voluntary and would not influence the planned procedure in any way. Recruitment continued until the target number of participants who had been discharged from OSC after outpatient surgery had been reached. The participants were not pre-screened for specific socio-demographic characteristics or for surgical procedure.

### Study procedure

**Fig. 1:**
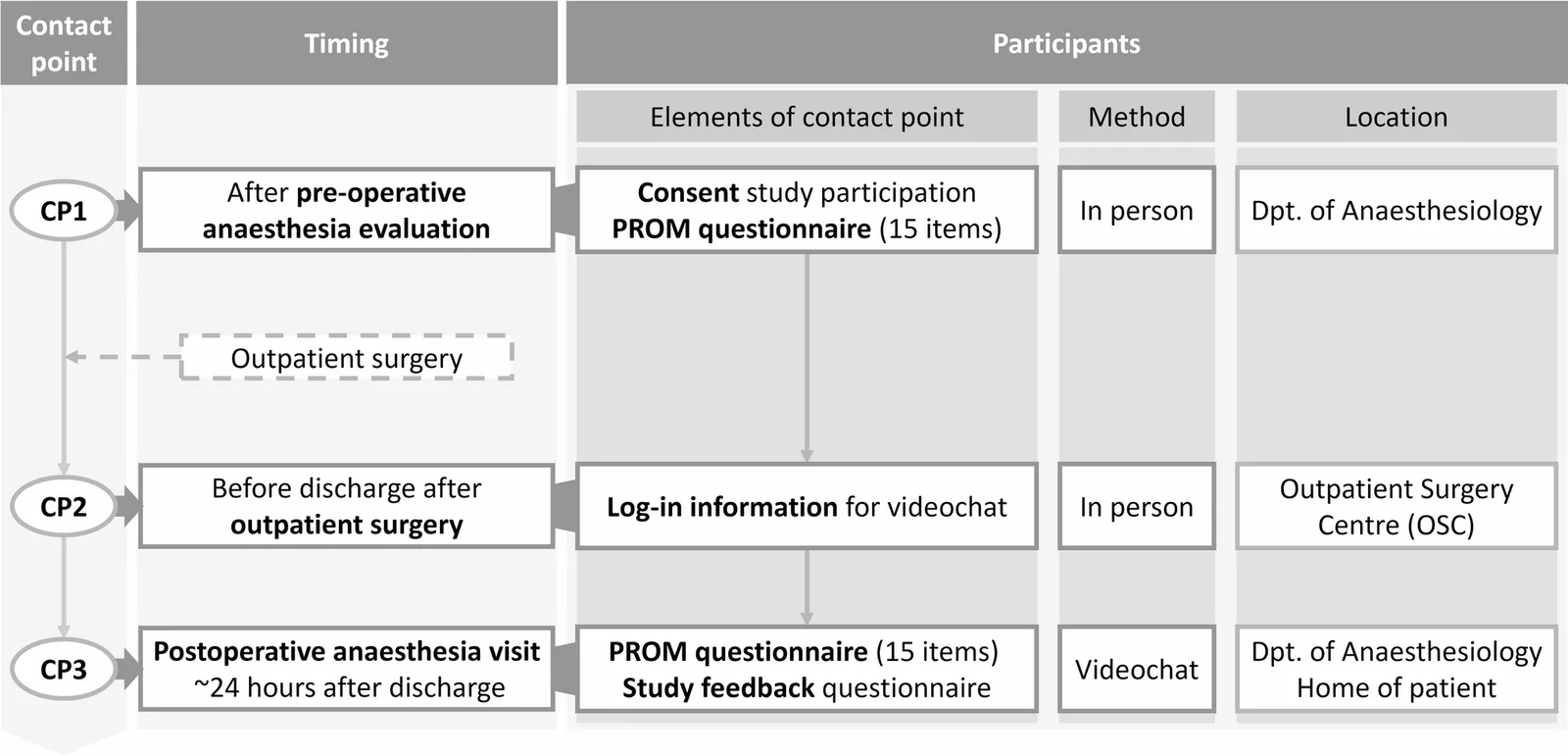
Contact points (CP) for observational pilot study. Participants were surveyed at two peri-operative contact points (CP1 and CP3) using PROM questionnaires. CP3 was conducted via videochat approximately 24 h after discharge

For the initial acceptance survey, a questionnaire with the items age, gender and four questions regarding perioperative care on the day of outpatient surgery was developed. These questions referred to the following: (1) satisfaction with the care received on the day of surgery, (2) fear of possible symptoms occurring after discharge, (3) feedback on the importance of immediate contact with a physician, and (4) evaluation of the possibility to use a videochat to clarify questions after discharge with the physician. Responses were collected using a four-point Likert scale, ranging from “strongly agree” to “strongly disagree,” with the additional option of “no answer.” The subsequent observational pilot study comprised three contact points (CPs) (Fig. 1). After pre-operative anaesthesia evaluation and written consent, participants completed a PROM questionnaire as first contact point (CP1) for pre-operative baseline assessment. The PROM questionnaires were based on the Quality of Recovery 15 (QoR-15) questionnaire (with the author’s permission). Each item was scored between 0 and 10 points, with 0 points representing a strongly negative and 10 points representing strongly positive feedback regarding the temporal presence of the respective items. The minimal clinically important difference (MCID) between two contact points was set at 8.0 points [13]. The four items ‘nausea or vomiting,’ ‘moderate pain,’ ‘severe pain,’ and ‘support from hospital doctors and nurses’ were selected as focus items for exploration for the observational pilot study representing typical postoperative issues from an anaesthesiologic point of view. Shortly before discharge (CP2), participants received written login instructions for the videochat as well as contact information for technical support. In the event of acute medical issues, participants were referred to the physician on duty and emergency services.

The third contact point (CP3) was the postoperative anaesthesia visit with an anaesthesiologist via videochat taking place approximately 24 h after discharge. The certified video consultation service TeleDoc (Docs in Clouds TeleCare GmbH, Aachen, Germany) was used for the videochat, allowing the physician and patient to hear and see each other. As the study was a pilot study focused on feasibility, the postoperative follow-up visit was not mandatory. Participants who did not participate were asked to provide brief feedback. In line with the study’s objective, an alternative follow-up was not conducted. The postoperative assessment was conducted with the PROM questionnaire from CP1. The interaction followed a standardized conversation script to rule out any acute need for medical intervention at the beginning of the videochat and standardize interaction. Following this, a questionnaire collecting patients’ perspectives on the observational pilot study with three main areas of focus was answered. Firstly, a comprehensive review regarding the implementation of the videochat was carried out covering prior participants’ experiences with videochats in the medical field, acceptance of the videochat in the outpatient setting and the comparison of videochats with phone calls. Secondly, the participants’ perspectives regarding the suitability and benefit of the PROM questionnaires in this context were gathered. Thirdly, participants provided feedback on using an app for answering the questionnaires and the acceptable time effort when no symptoms are present. If an immediate need for action would be identified at any point, a senior physician of the Department of Anaesthesiology was on call for assistance. Additional measures for follow-up besides the videochat at CP3 were not established in this study.

### Outcome parameters

The initial acceptance survey addressed the general relevance of a postoperative anaesthesia visit after outpatient surgery and willingness to use a videochat for that purpose.

The observational pilot study was designed to accompany the piloting of an optional postoperative anaesthesia visit via videochat after outpatient surgery as well as evaluating its utilisation rate. The videochat and PROM questionnaires were evaluated as synchronous and asynchronous tools in terms of their suitability and perceived benefit for aftercare and clinician-patient interaction from the patients’ perspective. Additionally, the study examined whether clinically relevant differences would justify a postoperative anaesthesia visit, despite the additional effort involved, and what factors might influence this. Furthermore, the study surveyed key points regarding how the postoperative anaesthesia visit should be conducted from the patient’s perspective.

### Study size

For the initial acceptance survey, we set a sample size of 300 adults (*n* = 300), representing a distribution across different age groups and accounting for individuals above the age of 70.

As part of the literature search, we assumed a 5.0% probability of relevant symptoms or technical issues occurring.^7^ According to Viechtbauer et al., we calculated that a minimum sample size of 59 individuals was needed to detect at least one negative effect or technical issue with 95% confidence [14]. For the observational pilot study, we defined a sample size of 100 participants (*n* = 100) to complete CP2, to account for the possibility that some participants would not attend the postoperative anaesthesia visit and were lost to follow-up. The statistical power in this study setting was not calculated based on the MCID as part of the main study with focus on the feasibility of combining videochat and PROM in the anaesthesiologic context.

### Statistical methods

The data were analysed using IBM SPSS Statistics for Windows, version 29 (IBM Corp., Armonk, NY, USA). The data are presented as number (%), median [IQR] and mean ± SD. To further assess the influence of age as possible impact concerning use of technologies like videochats, an analysis of age groups was conducted for the initial acceptance survey with age groups comprising five years. People older than 70 years were grouped [15].

The PROM questionnaire scores were not normally distributed according to the Kolmogorov-Smirnov test. Significance was considered with a p-value of ≤ 0.05 and analysed using the Mann-Whitney-U-Test, the Wilcoxon signed rank test or paired sample sign test if the requirements were not met. To compare the participants of the observational pilot study who completed all three contact points against the participants that did not, the distribution of gender was compared using χ2 test. In each case, age was normally distributed and the distribution of age was compared using the unpaired t-test.

## Results

### Results initial acceptance survey

**Fig. 2:**
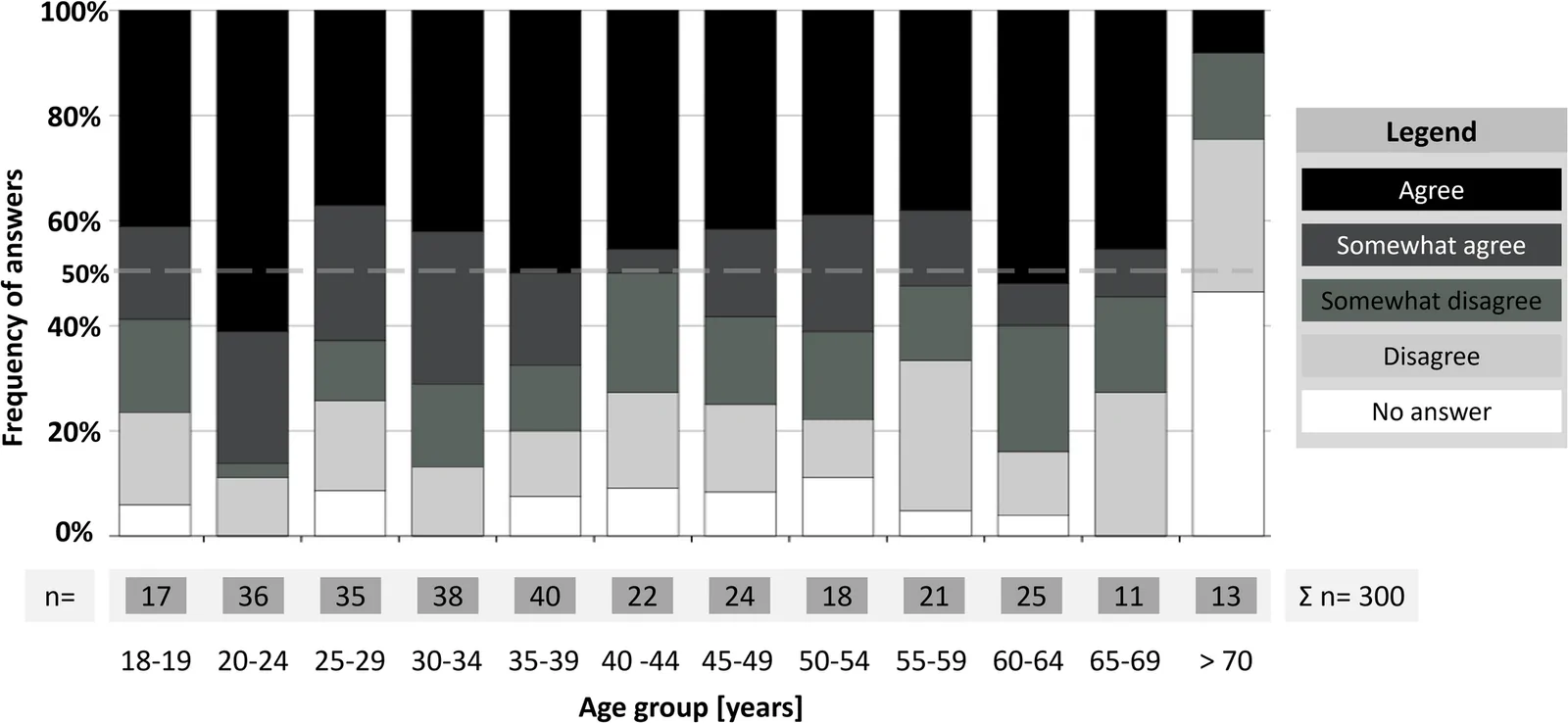
Initial acceptance survey item: "I might consider using videochat for questions after discharge". The survey responses were analysed by age group. Most of the feedback was positive in each group, except for the over-70 age group

The mean age of the participants in the initial acceptance survey was 40.8 years (SD 16.5, range 18 to 89 years). 59.7% of participants identified as female. In the initial acceptance survey, 99.3% of patients reported satisfaction with the care they received on the day of outpatient surgery. Although 64.7% of respondents stated that they were not concerned about pain or side effects after discharge, 74.0% indicated that it was of great importance to have immediate access to a physician when needed. Nearly two-thirds (62.0%) of respondents expressed support for the use of videochat to address any remaining concerns regarding anaesthesia after discharge. Men were more likely to support using videochats (65.3%) compared to women (59.8%). The majority of respondents in the under-70 age groups expressed support for the use of videochats, indicating either agreement or a somewhat agreement with this approach (Fig. 2). In contrast, there was only little agreement (7.7%) among respondents in the comparatively small age groups above 70 years.

### Results observational pilot study

**Fig. 3:**
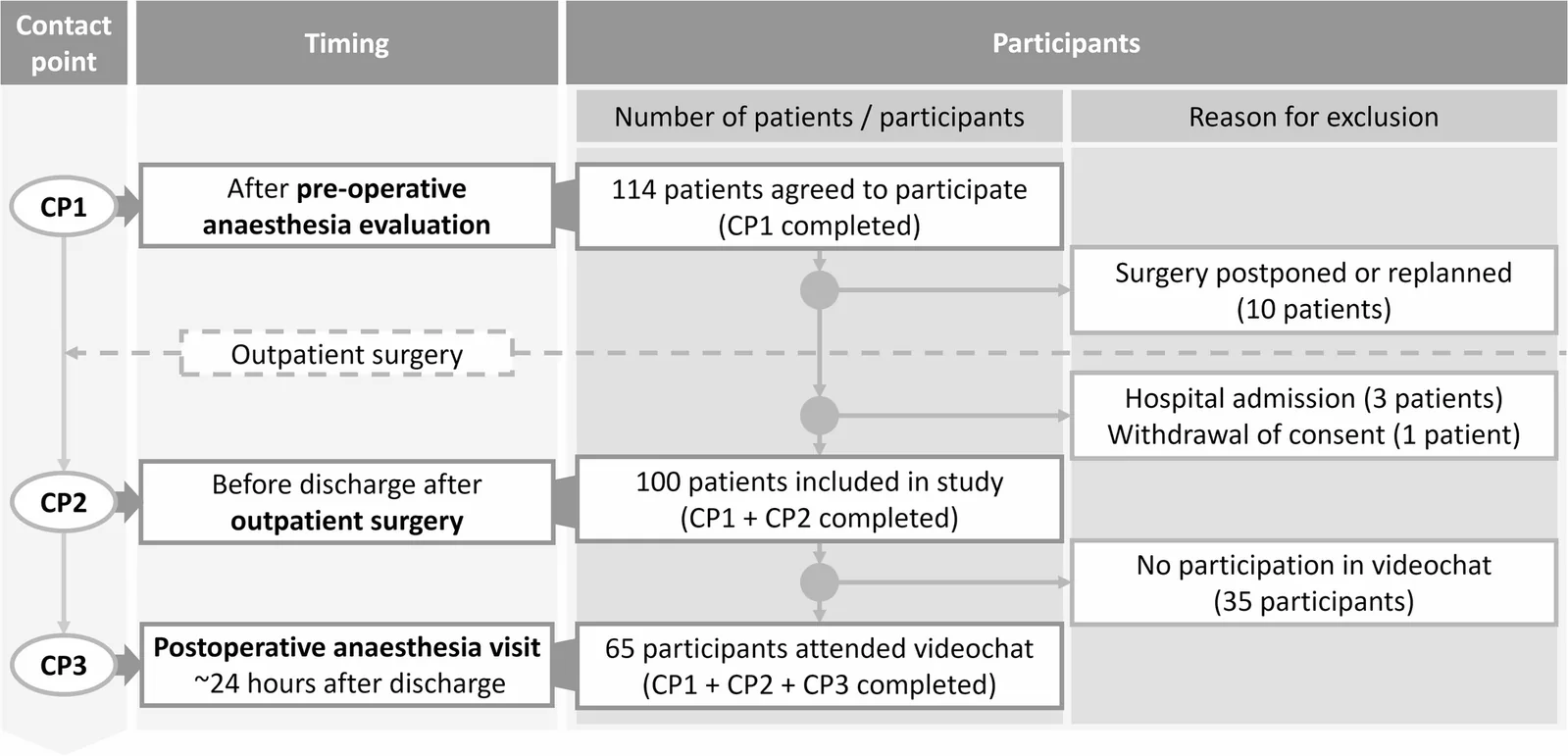
Study population of observational pilot study. Patients were excluded from the observational pilot study due to organizational, medical and individual reasons. The final study population consisted of 100 participants, 65 of whom attended the postoperative anaesthesia visit

A total of 114 patients were initially enrolled in the study. 14 patients were excluded because of postponed or rescheduled surgery (*n* = 10 patients), postoperative hospital admission (*n* = 3 patients) and withdrawal of consent (*n* = 1 patient) (Fig. 3). The mean age of the participants completing CP 1 and 2 (*n* = 100) was 37.5 ± 12.2 years (age range 19 to 67 years). 65.0% of participants identified as female.

Of the 100 respondents at CP2, 65 participated in the postoperative anaesthesia visit at CP3 and were included in the subsequent evaluation. Reasons provided for non-participation were attending the emergency department (*n* = 1), feeling well (*n* = 1) and having other appointments (*n* = 2). For the other participants who did not attend the videochat, no postoperative problems were reported to the study team. The mean age of the participants at CP3 was 39.3 ± 12.2 years (age range 19 to 67 years) with 70.8% of participants identifying as female. People completing all three contact points were on average older than people who did not (34.3 ± 11.9 years). There was a statistically significant difference between the ages of these two groups [t(98) = 1.99; *p* = .049; d = 0.42] with a higher mean age of 5.0 years (95%-CI[0.01, 10.09]). There was no difference in gender in those who also attended the postoperative anaesthesia visit and those who did not [χ²(1) = 2.72, *p* > .05, φ = -0.17].

Videochat participants showed significant different distributions for the items ‘getting support from hospital doctors and nurses’ and ‘moderate pain’ at CP1 [p = .036] than non-participants. Furthermore, significant differences regarding the item ‘severe pain’ could be detected at CP1 [p < .001]. The videochat participants showed, in comparisons, distributions that included lower scores. For the item ‘nausea and vomiting’ no significant difference could be found [p > .05] between videochat participants and non-participants at CP1.

**Fig. 4:**
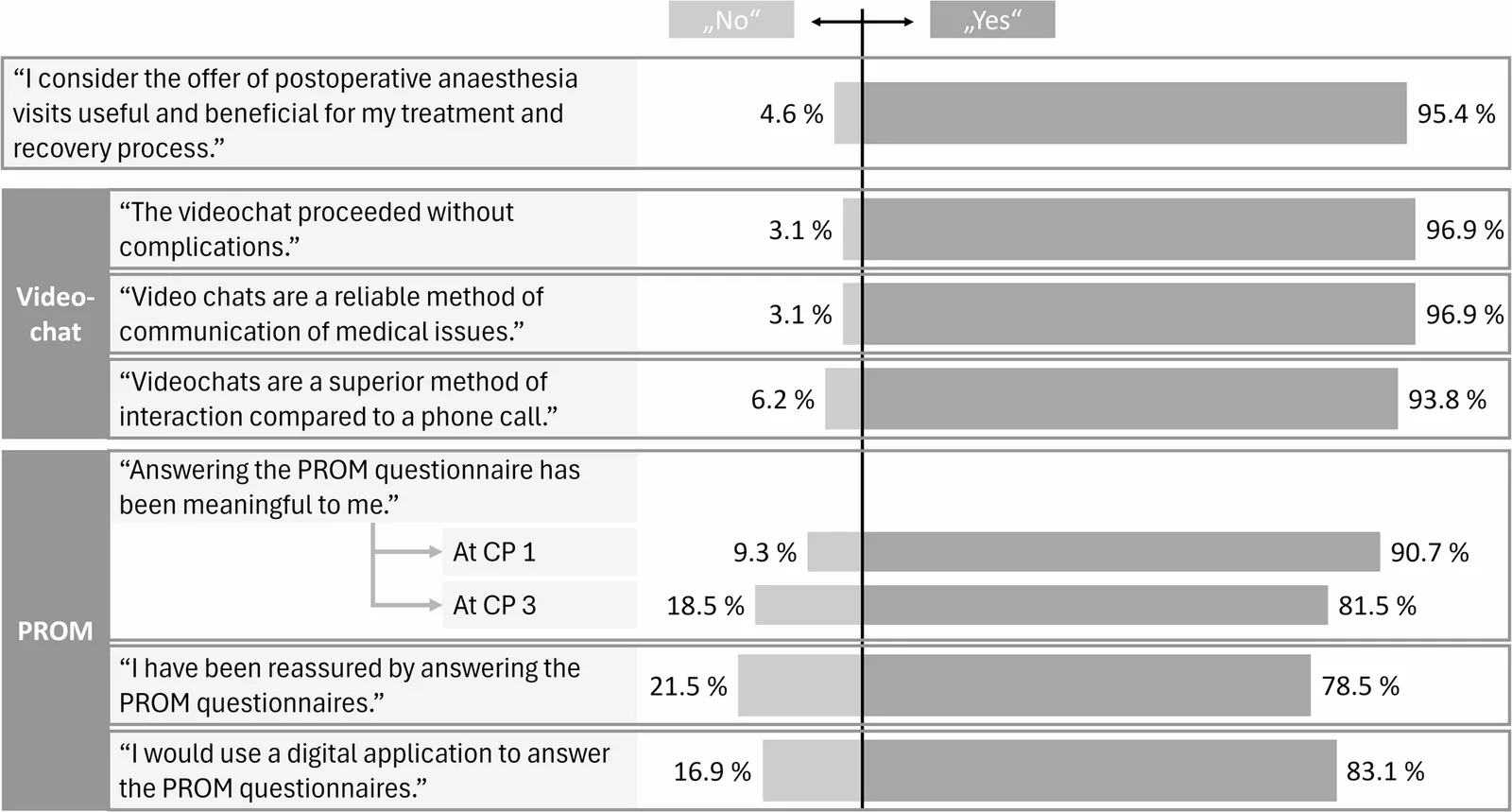
Feedback of participants regarding postoperative anaesthesia visit. Participants (n = 65) were surveyed at CP 3 regarding the offer of the postoperative anaesthesia visit and the implementation via videochat and PROM questionnaires

62 (95.4%) videochat participants found the offer of postoperative anaesthesia visits useful and beneficial for their treatment and recovery process. Technical support for camera and microphone accessibility issues and lost login information was required by 2 (3.1%) participants. 63 (96.9%) participants considered videochats as a reliable tool for discussing medical issues (Fig. 4). Furthermore, 61 (93.8%) participants perceived videochats as a superior method of interaction compared with phone calls. Videochats had been used before in a medical context by 28 (43.1%) participants.

Answering PROM questionnaires at CP1 and CP3 was considered meaningful with 90.7% agreement for CP1 and 81.5% agreement for CP3 respectively. 78.5% of participants gained reassurance through answering the questionnaires. There was no statistically significant difference in total PROM questionnaire scores between CP3 and CP1 [z = -0.8, *p*=.13]. Statistically significant differences were identified for ‘moderate pain’ [z = -2.6, *p*=.010] and ‘nausea or vomiting’ [z = -2.1, *p*=.033] but not for ‘severe pain’ and ‘support received from nurses and doctors. MCID for total PROM questionnaire scores was observed in 37 out of the 65 participants with 21 participants feeling worse at the postoperative evaluation. None of the participants was referred to a senior physician of the Department of Anaesthesiology. 54 (83.1%) of the 65 participants would use a digital application to answer the questions.

When asked how much time the respondents would spend on digital follow-up if symptom-free, 42 (64.6%) participants indicated that they would spend approximately fifteen minutes on the digital follow-up. 18 (27.7%) would like to spend less than 15 min and 5 (7.7%) participants would not like to limit the effort initially. The median duration of the videochats including jointly answering both questionnaires was 7.0 [6 to 13] minutes. 24.6% responded that they prefer a fixed appointment for the postoperative anaesthesia visit whereas 36.9% prefer to make an appointment at short notice when needed. 38.5% preferred to have both options. In addition to the videochat, the extra work for the hospital staff included scheduling the appointments and preparing log-in information, reviewing the questionnaire in advance and the subsequent documentation.

## Discussion

In the initial acceptance survey, nearly two-third of respondents stated they could imagine using videochat to answer questions after discharge. The subsequent observational pilot study implemented a postoperative anaesthesia visit after discharge using videochat and PROM questionnaires. Most participants considered this a beneficial part of anaesthesia care and rated videochats as superior to telephone calls. The PROM questionnaires were considered to improve perceived safety and as a meaningful addition to the peri-operative processes.

As the majority of patients stated interest in the possibility of using a videochat to discuss questions and problems arising from anaesthesia after discharge, we successfully piloted a postoperative anaesthesia visit. The findings of the pilot phase were consistent with those of the acceptance survey.

Almost two-thirds of participants in the observational pilot study chose to make use of the offer of a postoperative anaesthesia visit. The number of participants in CP3 exceeds the minimum number of participants required. At the same time, the loss to follow-up means that the responses received must be interpreted in this context. This could result in missing out on relevant feedback. Although more than half of the participants had never participated in a medical video consultation before, the few technical problems that were communicated could be resolved quickly. Previous systematic patient training was not required. Regarding non-participation, technical problems should be considered a minor factor given the availability of technical support. Particularly in the younger age groups there was a higher level of non-participation for the videochat although younger people showed high levels of interest for potential use in the initial acceptance survey. The presence of only mild symptoms, preference for selective discussion rather than a scheduled appointment as well as the decision to see a physician in person, could potentially have influenced the decision not to participate. Scheduled anaesthesia visits may conflict with personal scheduling, especially in cases of rapid recovery. Alternatively, complications may have arisen that made participation impossible.

As Kingery et al. stated, outpatient follow-up via videochat is considered superior to telephone calls from a surgical perspective [16]. This study indicates that this may also apply for the anaesthesia context. The importance of technical reliability was emphasized by the fact that the participants who experienced problems using the videochat did not consider videochat to be a reliable tool for medical issues.

The use of PROM questionnaires at three perioperative contact points revealed clinically relevant differences for participants in total and specifically for anaesthesiologic related items over time. Some participants did not regain their pre-operative status supporting the potential need of additional postoperative anaesthesia visits. Some participants used the offer of a postoperative anaesthesia visit despite not feeling worse than before surgery. The systematic, multifaceted coverage with PROM questionnaires was perceived a positive addition as it increased the patients’ perceived safety [17]. Patients are usually unable to assess complications that arise and need support for clarification. For technical reasons, the PROM questionnaires at CP3 were completed in this study in consultation with a physician. Asynchronous assessment via PROM questionnaires does not per se create additional work for physicians. Should physician-patient contact be necessary, the collected data supports the physician’s assessment and allows for a problem-oriented and targeted conversation. The more often a postoperative visit results in a patient no longer requiring further medical assistance, the more economically useful it becomes. For outpatient gynaecological procedures, the economic benefits of an app-based follow-up option were already confirmed [18]. The time needed for the PROM questionnaire and videochat with script after discharge is lower than the time patients were prepared to spend even if symptom-free. Consistent feedback enables a multifaceted outcome assessment and quality control of the growing number of outpatient cases from the perspective of peri-operative anaesthesia care.

### Limitations

The chronological inclusion of participants may have led to an overweighting of certain procedures or socio-demographic characteristics. In addition, more detailed descriptions of anaesthesiologic procedures and general circumstances, the severity of the procedure and the potential resulting complications such as pain were not recorded. This may have created the potential for selection bias and limited generalisability to the outpatient surgical population. Especially missing is the relationship between the type of surgery performed and the need for a postoperative anaesthesia visit. The observational pilot study did not include measures to follow up with participants not attending the videochat. Contact beyond the three designated contact points was not planned, creating bias in the feedback received. It was not possible to obtain potentially relevant feedback on clinical status or perceived benefit from these participants. The satisfaction rate may be biased towards those who were already amenable to the intervention and did not encounter complications hindering their participation. The videochat and completion of the questionnaires during CP 3 were conducted together with a physician from the Department of Anaesthesiology. Observer effects may have inflated positive ratings or allowed for social desirability bias. The follow-up period was limited in time with the potential of missing relevant changes in patients’ status.

### Outlook

The study demonstrated the feasibility of implementing a postoperative anaesthesia visit as well as its potential to be well received. Further research should focus on validating the results of the pilot study and, in particular, to demonstrate the comparative effectiveness from a medical, patient, and economic perspective. To support claims of benefits over standard or no follow-up, an extended study design should be adopted that takes into account readmissions, unplanned emergency department visits and general complications.

It would be beneficial to better understand the reasons for participation or non-participation to identify relevant factors and optimize the process. The cost-effectiveness mentioned in other studies should be confirmed further by identifying patient groups benefiting most from postoperative anaesthesia visits. Older patients tend to experience more complications and symptoms after outpatient surgery [19] and may be of particular interest in this context.

### Conclusion

The growing number of outpatient procedures and their expanded indications increase the number of self-determined recovery periods at home. The absence of direct support from medical professionals necessitates the implementation of new measures to assist with patient enquiries and prevent overburdening of the healthcare system. Postoperative anaesthesia visits after outpatient surgery using videochats and PROM questionnaires were successfully piloted for this study population and may be a feasible mean to support effective physician-patient communication after discharge. Study participants consistently perceived the offered videochat combined with PROM questionnaires as adequate and beneficial measures in the study setting. Telemedicine post anaesthesia visits may be an efficient component of patient treatment, helping to endeavour a differentiated and patient-centred care with limited resources. Further controlled studies are needed to assess the influence on postoperative safety and clinical outcomes as well as to better understand the influencing factors for the need for postoperative anaesthesia visits after outpatient surgery.

## Data Availability

The datasets used and analysed during the current studies are available from the corresponding author on reasonable request.

## Abbreviations

CP: Contact point
OSC: Outpatient Surgery Centre
PROM: Patient-related outcome measures
